# Grafting Animals

**Published:** 1864-12

**Authors:** 


GRAFTING ANIMALS.—Dr. Paul Bert has published a work on the curious subjects of animal grafts. He succeeded in making Siamese twins of a couple of rats, and many other monstrosities. He exclaims, "It is a surprising spectacle to see a paw cut from one rat, live, grow, finish its ossification, and regenerate its nerves under the skin of another, and when we want a plume of feathers under the skin of a dog, what a miracle to see the interrupted vital phenomena resume their course, and a fragment of a bird receive nourishment from the blood of a mammal.—*Intellectual Observer.*

